# Transversal gene expression panel to evaluate intestinal health in broiler chickens in different challenging conditions

**DOI:** 10.1038/s41598-021-85872-5

**Published:** 2021-03-18

**Authors:** L. Criado-Mesas, N. Abdelli, A. Noce, M. Farré, J. F. Pérez, D. Solà-Oriol, R. Martin-Venegas, A. Forouzandeh, F. González-Solé, J. M. Folch

**Affiliations:** 1 Plant and Animal Genomics, Centre for Research in Agricultural Genomics (CRAG), CSIC-IRTA-UAB-UB Consortium, Bellaterra, Spain; 2 Animal Nutrition and Welfare Service, Animal and Food Science Department, Facultat de Veterinària, Universitat Autònoma de Barcelona, 08193 Bellaterra, Spain; 3 Animal and Food Science Department, Facultat de Veterinària, Universitat Autònoma de Barcelona, 08193 Bellaterra, Spain; 4 Department of Mathematics, Area of Statistics and Operations Research, Universitat Autònoma de Barcelona, 08193 Bellaterra, Spain; 5 Department of Biochemistry and Physiology, Facultat de Farmàcia i Ciències de l’Alimentació, Universitat de Barcelona, 08028 Barcelona, Spain; 6 Research Institute of Nutrition and Food Safety (INSA-UB), Universitat de Barcelona, 08291 Santa Coloma de Gramanet, Spain

**Keywords:** Genetics, Molecular biology

## Abstract

There is a high interest on gut health in poultry with special focus on consequences of the intestinal diseases, such as coccidiosis and C*. perfringens*-induced necrotic enteritis (NE). We developed a custom gene expression panel, which could provide a snapshot of gene expression variation under challenging conditions. Ileum gene expression studies were performed through high throughput reverse transcription quantitative real-time polymerase chain reaction. A deep review on the bibliography was done and genes related to intestinal health were selected for barrier function, immune response, oxidation, digestive hormones, nutrient transport, and metabolism. The panel was firstly tested by using a nutritional/*Clostridium perfringens* model of intestinal barrier failure (induced using commercial reused litter and wheat-based diets without exogenous supplementation of enzymes) and the consistency of results was evaluated by another experiment under a coccidiosis challenge (orally gavaged with a commercial coccidiosis vaccine, 90× vaccine dose). Growth traits and intestinal morphological analysis were performed to check the gut barrier failure occurrence. Results of ileum gene expression showed a higher expression in genes involved in barrier function and nutrient transport in chickens raised in healthy conditions, while genes involved in immune response presented higher expression in *C.perfringens*-challenged birds. On the other hand, the *Eimeria* challenge also altered the expression of genes related to barrier function and metabolism, and increased the expression of genes related to immune response and oxidative stress. The panel developed in the current study gives us an overview of genes and pathways involved in broiler response to pathogen challenge. It also allows us to deep into the study of differences in gene expression pattern and magnitude of responses under either a coccidial vaccine or a NE.

## Introduction

The interest on the gut health in poultry has grown over the past two decades with special focus on consequences of the intestinal diseases. Pathogens may damage the intestinal mucosa, leading to impaired absorption of nutrients, reduced weight gain and decreased overall performance. In particular, the avian gut function is reported to be seriously threatened by coccidiosis^1^ and C*. perfringens*-induced necrotic enteritis (NE)^2,3^, especially under antimicrobial-free production systems. Thus, the high economic losses associated with poor feed efficiency, mortality, and medication costs have fuelled the search for biomarkers that likely associate with normal or abnormal conditions^4^, and allow the understanding of events affecting the intestinal barrier, its functionality, and the ecology of the gastrointestinal microbiota^5^. Previous studies have reported that intestinal gene expression of mucins, tight junctions (TJ) and nutrients transporters may be considered as gastrointestinal biomarkers of intestinal barrier function (BF)^1,5^. It has also been reported that inflammation associated with oxidative stress (OX) may produce physiological changes in gene expression which suggests that inflammation-induced OX plays a crucial role in intestinal function^1,6^. Thus, we hypothesized that gut health challenges may induce changes in the intestinal gene expression, and that the overall assessment of expression levels of a wide range of genes involved in several functions, such as BF, immune response (IR), and OX, among others, may provide an overall insight into the host responses during coccidiosis and NE challenges. Therefore, our objective was to develop a custom gene expression panel, which could provide a snapshot of gene expression variation under challenging conditions. Hence, a deep review of the publications related to this topic was carried out, and a list of candidate genes that act as markers in a wide range of functions in the intestine were selected, including the BF, IR, OX, digestive hormones (H), nutrient transport (NT), and metabolism (MB). The panel was firstly tested by an experiment using a nutritional/*Clostridium perfringens* model of intestinal barrier failure and the consistency of results was further evaluated by another experiment under a coccidiosis challenge where the protein expression of *OCLN* and *CLDN* was also measured.

## Results

### C.perfringens challenge

#### Growth performance

Supplementary Table 1 shows that *C.perfringens*-challenged chickens exhibited lower body weight (BW) on day (d) 10 (*P* = 0.04), 28 and 42 (*P* < 0.001) as a result of lower average daily gain (ADG; *P* < 0.001) compared to the group raised in cages. The overall feed conversion ratio (FCR) was significantly increased (*P* < 0.001) in challenged birds (2.07) compared to the non-challenged group (1.54).

#### Intestinal morphological analyses

As shown in Supplementary Table 2, chickens raised in cages presented higher villus height (VH; *P* = 0.016), reduced crypt depth (CD; *P* < 0.001), improved VH:CD ratio and lower intraepithelial lymphocytes/100 µm VH (*P* < 0.001) than those under *C.perfringens* challenge.

#### Intestinal gene expression

Due to technical problems, *AvBD9* gene was withdrawn from the study because of its low mRNA expression levels in all samples. Hence, a gene expression panel of 44 target genes was used in the current study.

**Figure 1:**
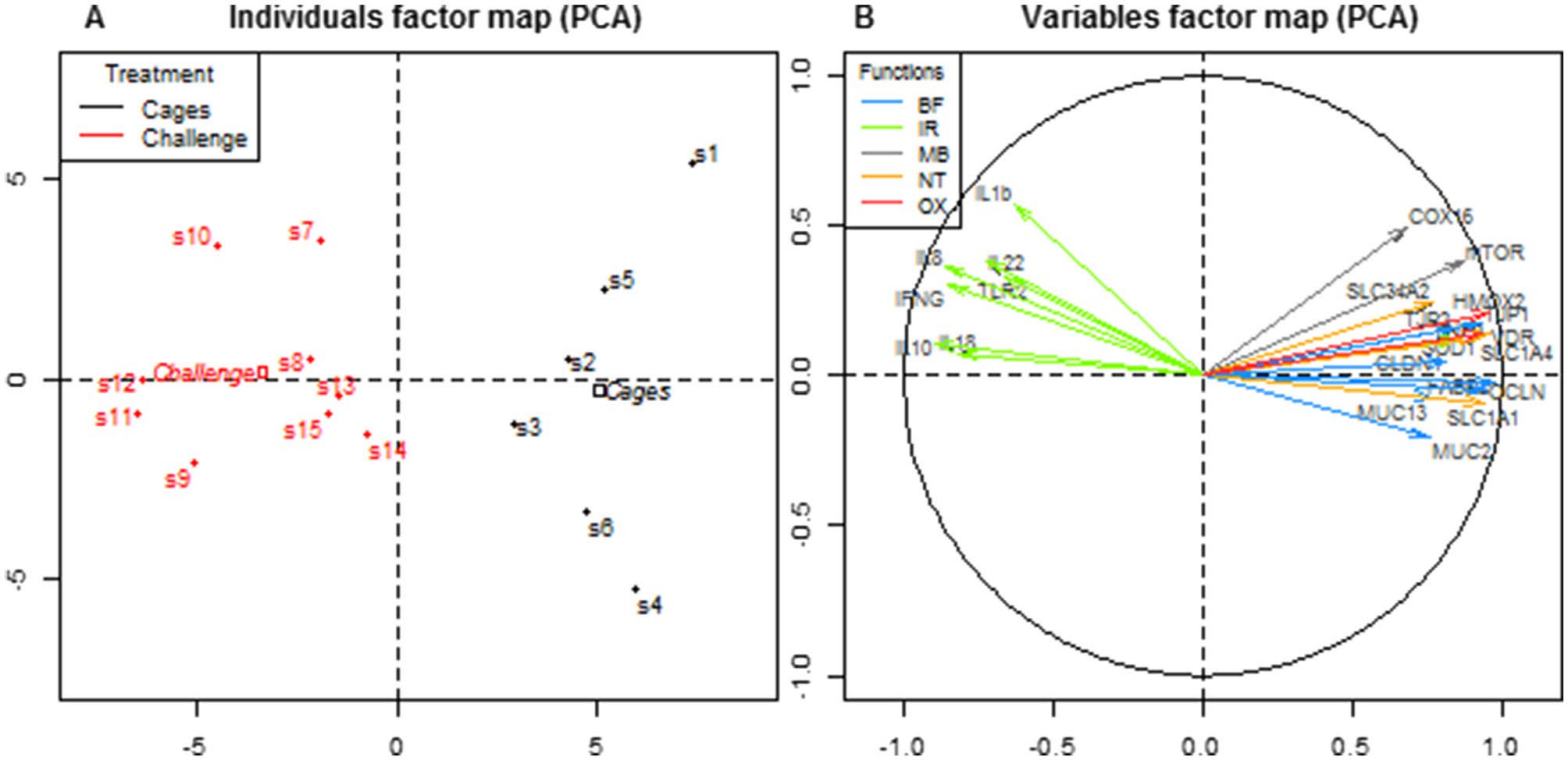
PCA representations of intestinal gene expression data in ileum chicken broiler samples (**A**) The samples dot plot; (**B**) variables arrow plot.

**Figure 2:**
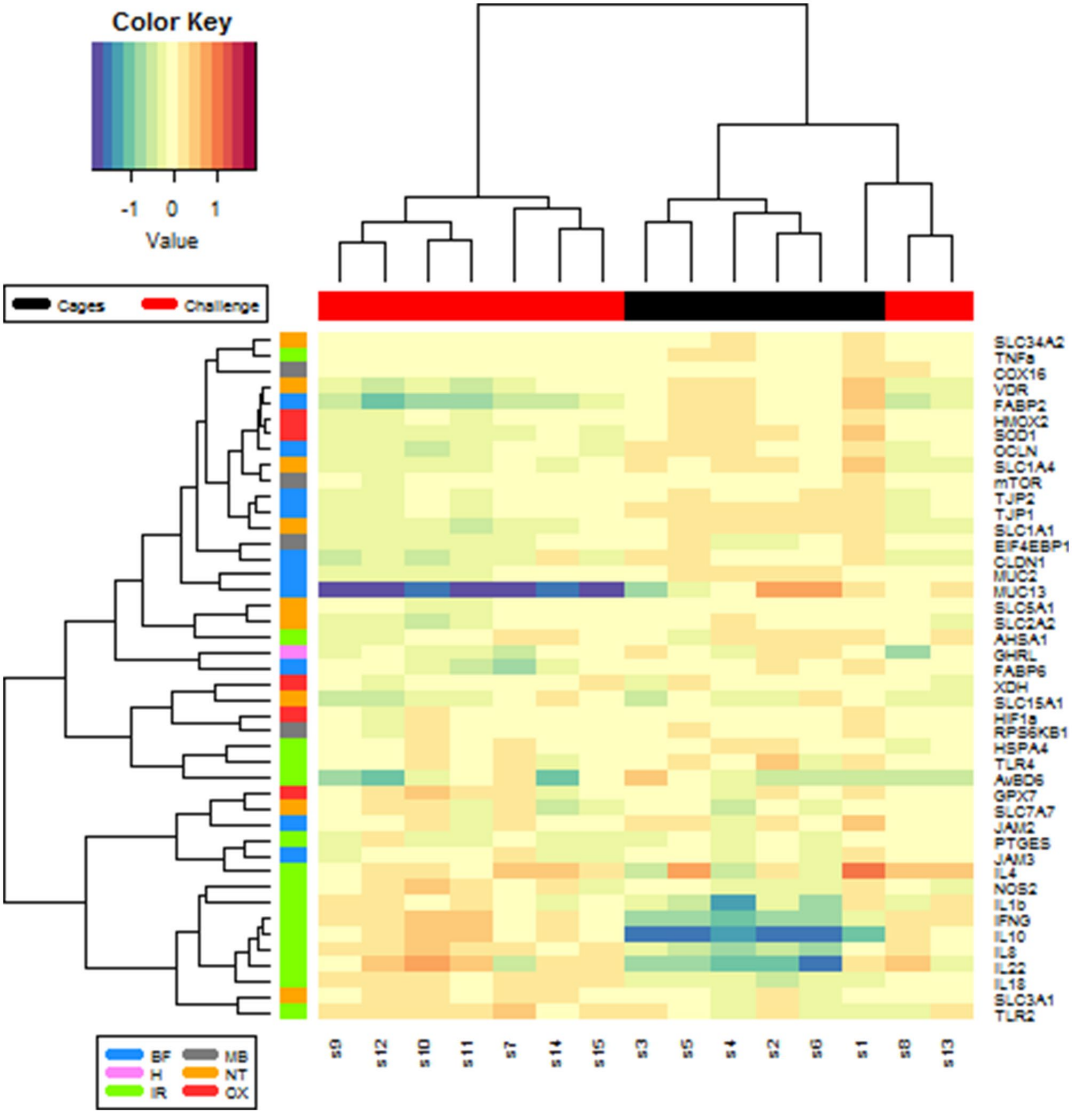
Heatmap of gene expression level of the 44 genes analysed in ileum. Genes were represented in the y-axis and samples in the x-axis. Experimental conditions (x-axis) were labelled in black colour for cages and red colour for challenging conditions. Groups of functions (y-axis) were labelled with different colours.

A principal component analysis (PCA) was performed with genes as variables and samples as cases with treatment as a qualitative variable, getting a two-dimensional (2D) representation that preserves the 62% of the total variance in the experiment. The samples dot plot (Fig. 1A) shows that the first principal component (46.36% of variance explained) separates the treatments: all the samples collected from chickens in cages are located on the right-hand side and the challenge samples on the left, with the squared points representing the means of the treatments clearly separated too. Although all the genes were used to define the principal dimensions, the variables used in the arrow plot (Fig. 1B) were restricted to the genes which showed significant expression differences between treatment means, and are well explained in the 2D-principal components space. This arrows plot combined with the samples dot plot showed a higher expression of the IR genes in chickens maintained under challenging conditions, while BF, MB, NT, and OX genes were more expressed in birds kept in cages. Moreover, a heatmap analysis showed patterns which are consistent with the PCA clusters (Fig. 2), in terms of sample expression pattern. Ileum samples were clustered each with their group (cages and challenge conditions), except for two samples. Genes were not grouped perfectly according to their function; however, IR genes tended to group together.

ANOVA-one-way was applied to the data of mRNA expression levels. The *P*-values obtained were adjusted for multiple testing using Benjamini–Hochberg procedure to control false discovery rate (FDR ≤ 0.05)^7^ and the output is summarized in Supplementary Table 3. All genes related to BF, except *JAM2* and *JAM3* genes, showed a higher expression in cages group than challenging one, with significant differences among them. *FABP6* (*P* = 0.08) and *GHRL* (*P* = 0.06) genes tended to be more expressed in cages group. Regarding the IR genes, *TNFa* (*P* = 0.001) presented higher gene expression values and *HSPA4* (*P* = 0.08) tended to be higher in cages group, whereas *IFNG*, *IL10, IL18*, *IL8 (P* < *0.001)*, *TLR2 (P* = *0.04)*, *IL22 (P* = *0.002)*, *IL1b* (*P* = 0.02) were more expressed in *C.perfirngens*-challenged chickens. In the MB function group, *mTOR* revealed higher gene expression (*P* = 0.002) while *RPS6KB1* and *COX16* showed a tendency to be more expressed (*P* = 0.089 and *P* = 0.057, respectively) in the cages group. For NT, *SLC1A1*, *SLC1A4 (P* < 0.001*)*, *VDR (P* = 0.001*)*, *SLC34A2* (*P* = 0.02) presented significantly higher gene expression levels and *SLC2A2* tended to be more expressed (*P* = 0.09) in chickens raised in cages. Finally, *SOD1* and *HMOX2* involved in OX showed higher mRNA levels (*P* < 0.001) in the cages group.

### Eimeria challenge

#### Growth performance

As shown in Supplementary Table 4, before *Eimeria* challenge (d7 and d9) no dietary treatment effect was observed on BW. After challenge, broiler chickens supplemented with the coccidostat showed higher BW15 (*P* < 0.001) than the non-supplemented NC group.

#### Coccidia oocyst shedding

Results of oocyst counting in the excreta of chickens on d 15 are reported in Supplementary Fig. 1. Broiler chickens supplemented with coccidiostat showed significantly reduced oocyst counting (*P* < 0.001) than the non-supplemented NC group.

#### Intestinal gene expression

**Figure 3:**
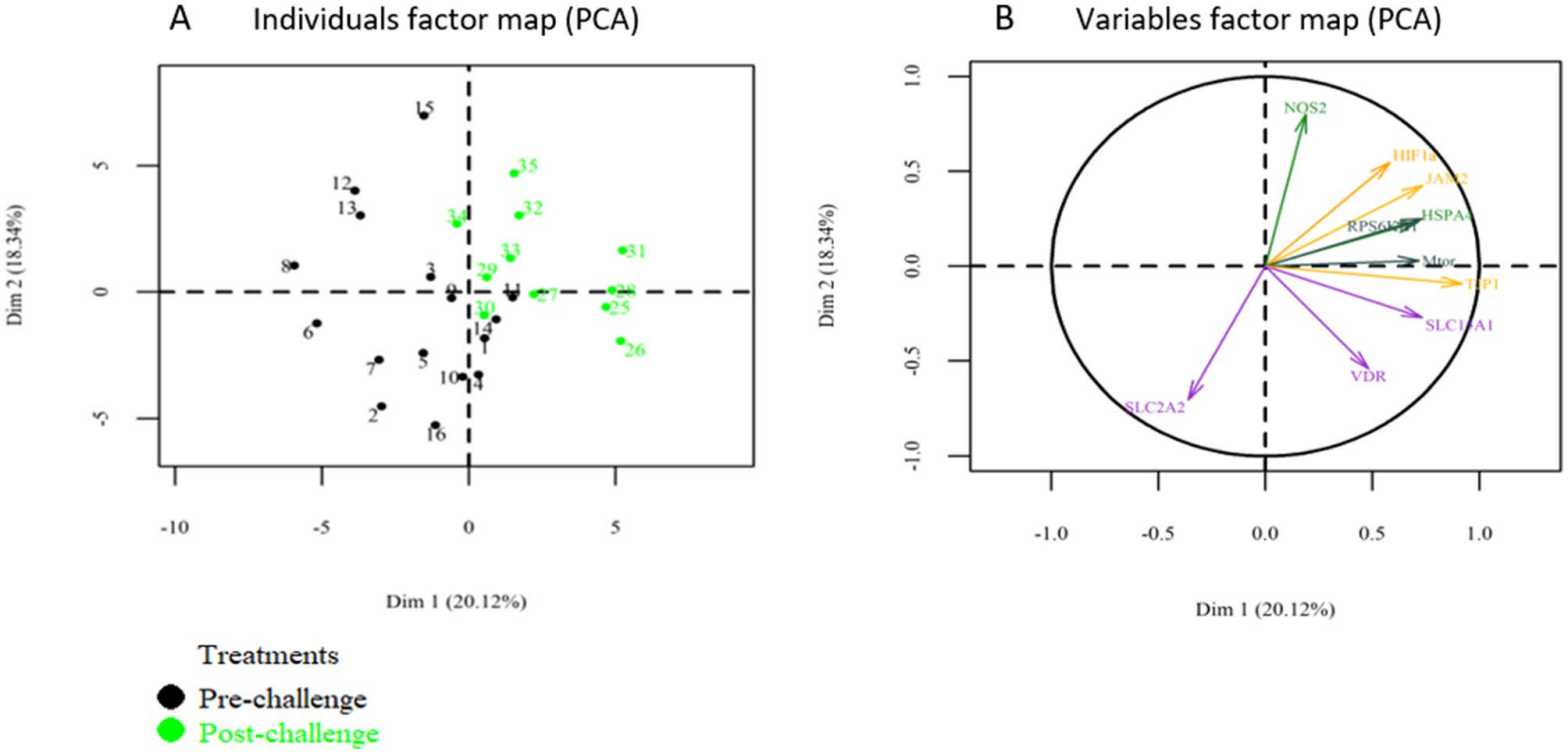
PCA representations of intestinal gene expression data in ileum chicken broiler samples (**A**) The samples dot plot; (**B**) variables arrow plot.

**Figure 4:**
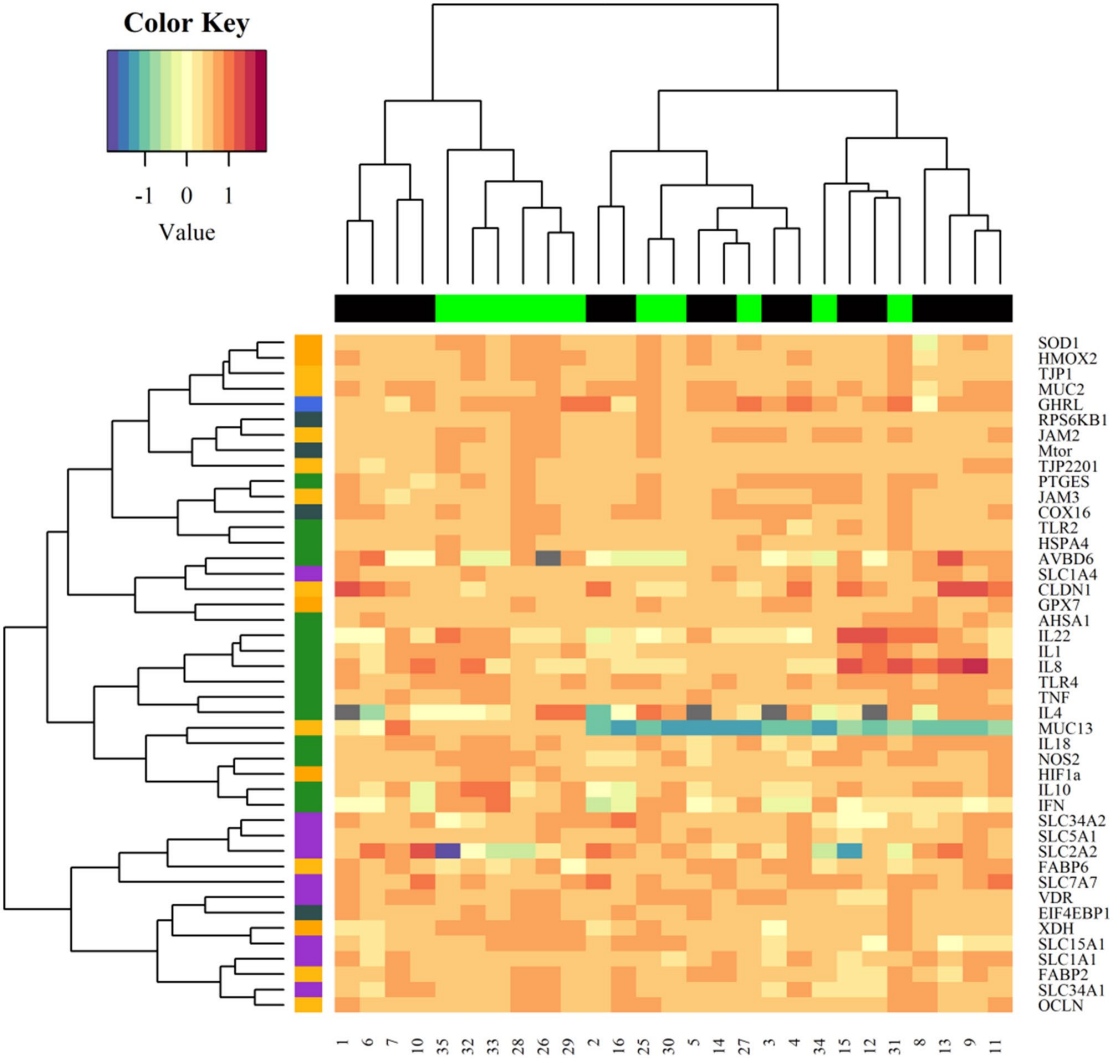
Heat map. The X-axis is sample expression pattern in different treatment group (black colour: before *Eimeria* challenge; green colour: after *Eimeria* challenge). The Y-axis is the gene clusters according to functions.

A first PCA was performed to observe the effect of *Eimeria* challenge on samples clustering. Although there was no perfect separation, the samples dot plot (Fig. 3A) showed that most samples of non-challenged chickens are located on the left-hand side whereas, those of *Eimeria* challenged birds are on the right. The variable arrow plot (Fig. 3B) did not show a clear separation between both groups according to gene function. Moreover, the expression pattern and gene clusters showed in the heat map (Fig. 4) were consistent with the PCA result, in terms of sample expression pattern. Neither the samples nor the genes were perfectly clustered according to the experimental group or function, respectively. However, IR genes tended to conglomerate together in the same group. Differences of gene expression were also examined between both groups and results are reported in Supplementary Table 5. The *Eimeria* challenge affected the expression of genes related to BF by reducing mRNA levels of *CLDN1* (*P* = 0.007) and *FABP6 (P* = 0.01*),* while increasing *JAM2* (*P* = 0.019) and *TJP1* (*P* = 0.014). It also increased the expression of *HSPA4* (*P* = 0.013), *INFG* (*P* < 0.001) and tended to increase *NOS2* (*P* = 0.07) related to IR. Regarding MB, *RPS6KB1* was significantly increased (*P* = 0.03) and *mTOR* tended to be increased (*P* = 0.07) by the *Eimeria* challenge. The challenged chickens also showed an increased level of *HIF1a* (*P* = 0.01) and *XDH* (*P* < 0.001), and a tendency to increase *SOD1* (*P* = 0.09) gene expression. Finally, an increase of *SLC15A1* (*P* = 0.007) and a tendency to decrease *SLC2A2* (*P* = 0.06) gene expressions was observed.

**Figure 5:**
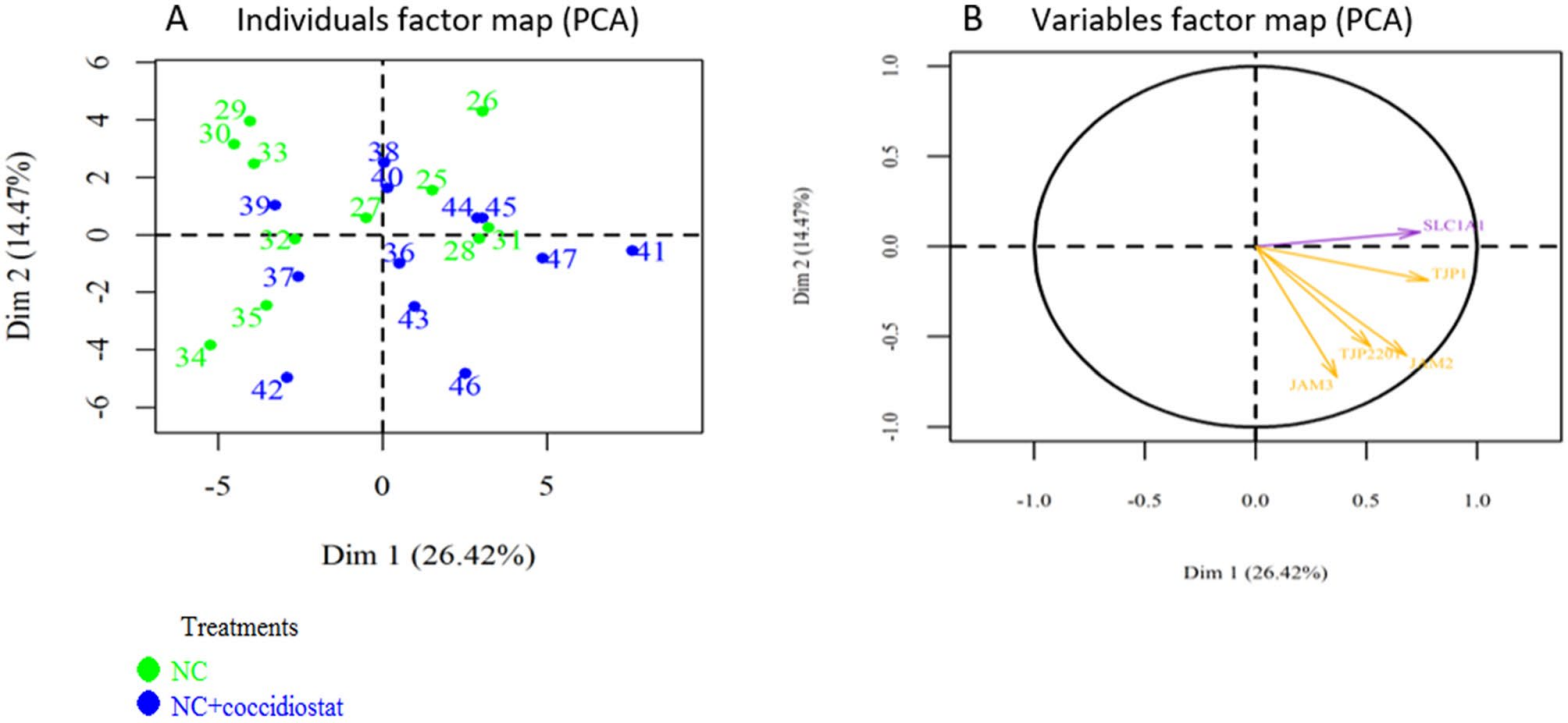
PCA representations of intestinal gene expression data in ileum chicken broiler samples (**A**) The samples dot plot; (**B**) variables arrow plot.

**Figure 6:**
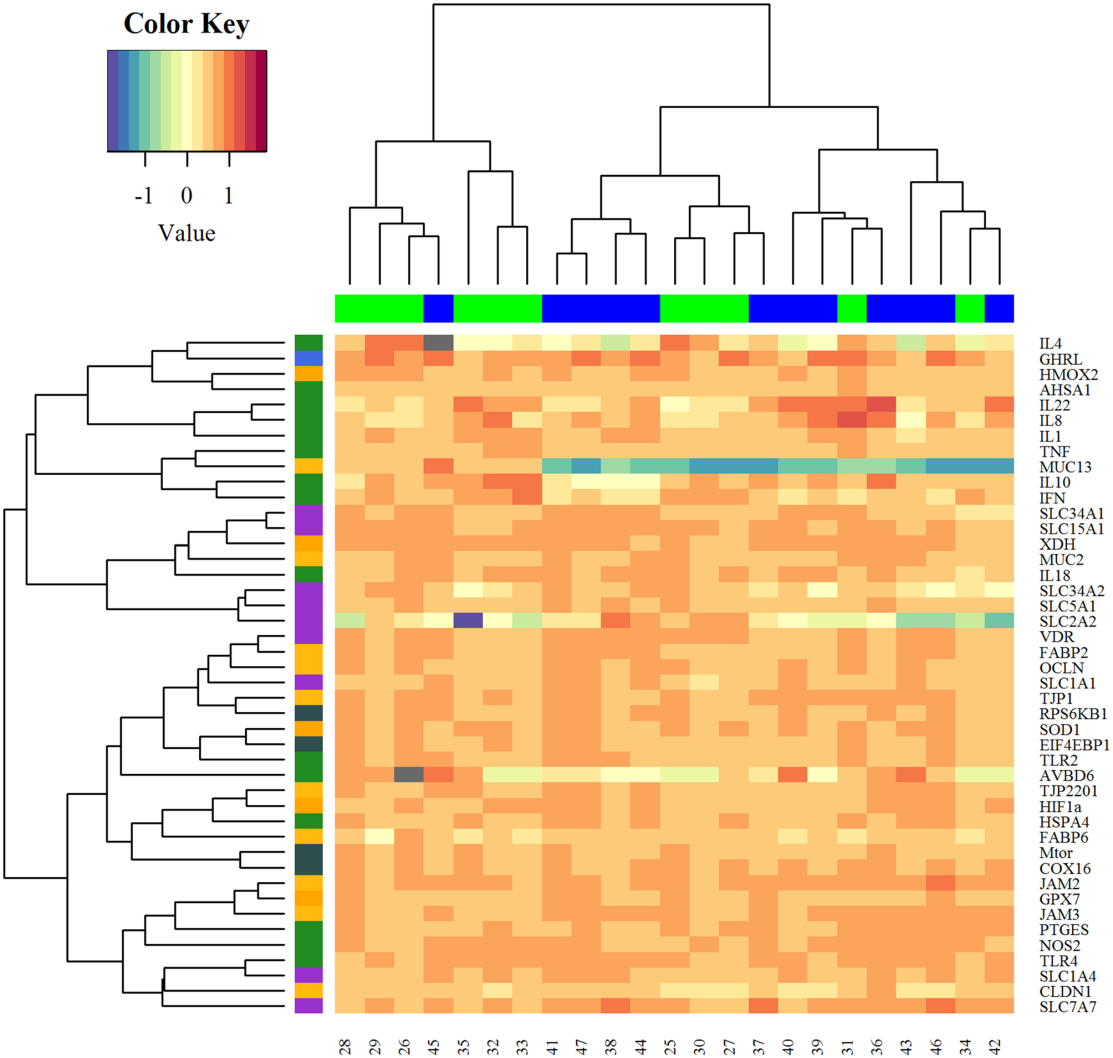
Heat map. The X-axis is sample expression pattern in different treatment group (green colour: negative control; blue colour: negative control + coccidiostat). The Y-axis is the gene clusters according to functions.

A second PCA was performed to study the effect of dietary coccidiostat supplementation on samples clustering. No clear separation was observed between samples collected from both groups (samples dot plot; Fig. 5A). The variable arrow plot (Fig. 5B) did not show a clear separation between both groups according to gene function. The results of a heat map developed to investigate the expression pattern and gene clusters (Fig. 6) were consistent with the PCA result in terms of sample expression pattern. Neither samples, nor genes were perfectly clustered according to the experimental group or function, respectively. Differences in gene expression were examined between both groups and results are represented in Supplementary Table 6. Supplementation of coccidiostat significantly reduced the mRNA levels of *INFG* (*P* = 0.006) gene which is related to IR, while two genes involved in NT, *SLC1A1* and *SLC7A7* (*P* = 0.07), tended to increase their mRNA expression levels.

#### Protein expression

As shown in Table 1, *Eimeria* challenge significantly reduced *CLDN1* protein expression (*P* < 0.001) but not the *OCLN* protein level (*P* = 0.33).

**Table 1:** Effect of *Eimeria* challenge on ileal *OCLN* and *CLDN1* protein expression (SEM: standard error of mean).

Protein (g/ng mucosa)	Experimental treatments		SEM	*P* value
	NC before challenge	NC after challenge		
*OCLN*	29.8	28.8	4.23	0.33
*CLDN1*	31.1	22.7	2.69	< 0.001

However, the supplementation of coccidiostat did not affect neither *CLDN1* nor *OCLN* protein level (Table 2).

**Table 2:** Effect of coccidiostat supplementation on ileal *OCLN* and *CLDN1* protein expression (SEM: standard error of mean).

Protein (g/ng mucosa)	Experimental treatments		SEM	*P* value
	NC	NC + coccidiostat		
*OCLN*	28.8	31.0	2.35	0.26
*CLDN1*	22.7	24.2	2.98	0.29

## Discussion

In the present study, the diet/*C. perfringens* challenge was induced by allocating newly hatched broiler chicks on a recycled litter characterized for its high content of *Clostridium perfringens,* as previously reported by^8^, and including wheat in the diet without xylanases, with the aim of increasing digesta viscosity and accentuating the dietary challenge. These broiler chickens showed a decrease of BW at d42 by 32% and an increase of global FCR by 26% compared to non-challenged birds. As for the *Eimeria* challenge, it resulted in a reduction of a 30% of BW at d15 compared to the standard Ross 308 values^9^. The compromised growth performance of *C.perfringens*-challenged chickens may be attributed to the damage of intestinal morphology as proven by the decreased VH:CD ratio suggesting a reduction of the absorptive capacity of the intestine^10^. This reduced ratio resulted from the decrease of VH and increase of CD indicating an increase of metabolic cost of intestinal epithelium turnover. The structural changes of the intestinal morphology induced by NE in the current study were in concordance with several authors^3,10^ and suggest a gut barrier failure occurrence which was later confirmed by the down-regulation of genes involved in NT and BF associated to an up-regulation of IR genes. The *Eimeria* challenge also affected the gene expression although to a lesser extent than the obtained with diet/*C. perfringens* diet, thus confirming the difference of the severity of the caused damage and demonstrating the adaptability of the gene expression panel to different challenge conditions.

### Nutrient transport

Our results showed a down-regulation of *SLC1A1* and *SLC1A4* mRNA levels of the *C.perfringens* challenged birds suggesting a reduced uptake of energy source glutamate and essential amino acids, respectively^11^, which may explain the major changes observed on the intestinal microvilli morphology and growth performance. Previous studies have also showed that *Eimeria* challenge leads to a decrease of *SLC1A1* gene expression^12,13^. However, in the current study no effect was observed on the *SLC1A1* gene expression suggesting that intestinal cells of the *Eimeria* challenged chickens did not suffer a depletion of the energy source glutamate. The mRNA levels of *SLC2A2*, located at the basolateral membrane of the gut epithelium and involved in glucose efflux, were reduced by the *Eimeria* challenge in the current study, which is in agreement with other authors^14,15^. However, our results showed an increase of ileum *SLC15A1* mRNA levels of *Eimeria*-challenged birds, which was not in concordance with results obtained by Miska and Fetterer^14^. This inconsistency may be attributed to the sampling site (ileum *vs* jejunum). In agreement with this hypothesis, Su et al.^12^ reported that *SLC15A1* mRNA levels were decreased in the jejunum and increased in the ileum of *Eimeria acervulina*-challenged layers. *C.perfringens*-challenged chickens showed a down-regulation of *SLC34A2* gene, which is involved in regulating both intestinal Pi (phosphate) absorption and renal Pi resorption, and of *VDR* gene, a transcription factor that mediates the vitamin D3, involved in signalling intestinal calcium and phosphate absorption^16^. This reduction of *SLC34A2* may be explained by the decrease of *mTOR*, known to stimulate many intestinal NTers including *SLC34A2*^17^*.* This down-regulation of *mTOR* may be attributed to the reduced amino acids and energy uptake in intestinal cells of *C. perfringens*-challenged broilers^18^ and the reduced VH:CD ratio of *C. perfringens*-challenged broilers. The *mTOR/RPS6KB1* pathway is essential to the intestinal cell migration^19^ and thus, could help to accelerate the healing of NE-induced intestinal damage and promote the recovery of tissues. In contrast, *Eimeria*-challenged birds showed an activation of *mTOR c*omplex 1 pathways indicating a higher gut cell turnover to reduce the intestinal mucosal disruption.

### Barrier function

Effects on TJ are pathogen-dependant as while some pathogens can utilize these proteins as receptors for attachment and subsequent internalization, others modify or destroy them by different pathways and thereby provide a gateway to the underlying tissue^20^. Accordingly, our results showed that TJ are differently modulated with either *C.perfringens* or *Eimeria* challenge. In general, *C. perfringens*-induced subclinical NE downregulated the gene expression of *OCLN, CLDN1, FABP2, TJP1,* and *TJP2,* and there was a tendency in the downregulation of *FABP6.* Meanwhile, *Eimeria* challenge showed a downregulation of *CLDN1* and *FABP6* and an upregulation of the *JAM2* and *TJP1* mRNA levels, as well as a reduction in *CLDN1* protein expression. *C.perfringens* enterotoxin (CPE) has been reported to use claudin family proteins within the tight junction structure as binding sites/receptors and eventually cause pore formation in host cells as well as an increase in paracellular permeability and cytotoxicity as a result of this attachment^21,22^. In fact, the decrease of intestinal *CLDN1*^23,24^, and *OCLN* mRNA expression^23,25–28^ has already been described in *C. perfringens*-challenged birds. However, the present study demonstrates that this challenge, not only causes a disruption in transmembrane proteins, but also in cytosolic protein that contribute to the functionality of TJ. Moreover, *MUC2*, a fundamental component of the protective mucus layer which can protect the intestine against pathogens and promote tissue restitution^29^, was downregulated by *C. perfringens*-induced subclinical NE, as observed by other authors, highlighting that the effects of this challenge were able to affect different components of the BF. In the case of the *Eimeria* challenge, the literature reported inconsistent results. As described by Soutter et al.^30^, the variability in the results could be related to the magnitude and timing of the challenge dose and vaccine formulation, as well as, chicken breed or genetic line. However, gene expression and the subsequent confirmation by protein expression that was performed in our study clearly demonstrate the participation of CLDN1 while seems to rule out the participation of OCLN in *Eimeria* challenge.

### Immune response

The binding mechanism of *C. perfringens* to the intestinal epithelial cell starts by the recognition and binding of pathogen-associated molecular patterns (PAMPs) by highly conserved pathogen recognition receptors, of which Toll-like receptors (TLRs) are the best characterized^31^. In particular, intestinal *TLR2* has been reported to recognize peptidoglycans in Gram-positive bacteria such as C.*perfringens,* and was upregulated in *C. perfringens* challenged broiler chickens in the current study, which corroborated the results of previous studies^23,32^. In the present work, *Eimeria* challenge showed no effect on *TLR2* and *TLR4* gene expression, which was in concordance with previous studies^33,34^. However, other authors reported that *TLR4* expression was upregulated at 12 h post infection (hpi) but not at 72 hpi in the ceum of *E.tenella* infected chickens^35^. Hence, this may explain why *TLR2* is not associated with chicken response to *Eimeria* infection, and suggest that *TLR4* is involved only in an early phase of response. Interactions between bacterial PAMPs and TLRs cause a cascade of signalling events that culminate in the secretion of various cytokines which have been reported to represent key components of innate immunity during the early phase of the host response to pathogens^36^. In the current study, we obtained an up-regulation in the *C. perfringens*-challenged group of *IL18, IL1B*, *IL22,* and *IL8.* These results indicated that *C. perfringens*-induced subclinical NE triggered an inflammatory IR in broiler chicken intestine as most pro-inflammatory cytokines were up-regulated. In this sense, the *IL8,* a CXC chemokine involved in the recruitment of leucocytes to mucosal sites of inflammation, was upregulated by *Eimeria maxima/C. perfringens* co-infection^37^ and *C. perfringens* infection alone^27^ or associated to high fishmeal diet^38^. Previous studies have also showed an up-regulation of *IL1B* in *C. perfringens* challenged broilers^2,23^. Moreover, both challenges significantly increased the proinflammatory cytokine *IFNG* mRNA levels compared with those in the unchallenged control, as previously reported in *C. perfringens*-^27,39^, *Eimeria spp-*^40^ and *C.perfringens/Eimeria maxima*-coinfected chickens^24^. The supplementation of coccidiostat resulted in a down-regulation of *IFNG* suggesting a reduced intestinal inflammation due to its ability to clear *Eimeria spp* as proven by the reduced oocyst shedding. *IFNG* has been also reported to affect the intracellular replication of *Eimeria spp* through the activation of inducible nitric oxide synthase (*iNOS*), an enzyme responsible for the production of nitric oxide (NO) proposed to be the effector molecule against *Eimeria spp*^41^. This may explain the tendency to upregulate mRNA levels of *2NOS* observed in the current study 6 d post infection (dpi) with *Eimeria*. In the case of *C. perfringens* infection, no effect on iNOS gene expression was observed, in contrast to other studies^42,43^. However, it has been reported that *iNOS* regulation is independent of *IFNG* during *C. difficile* colitis^44^, suggesting that the mechanism underlying this activation needs to be further investigated.

To protect the intestinal integrity, a release of anti-inflammatory cytokines would be expected to be observed in *C.perfringens*-infected birds. Our results are in agreement with other authors that reported an increase of *IL10* in *C. perfringens*^43^ and *C. butyricum* challenged birds^45^*.* However, a lack of response on *IL4* was observed in the current study and could be attributed to the age of chickens (42 d). Accordingly, Collier et al.^46^ showed an increased gene expression of *IL4* in birds co-infected with *Eimeria* and C. *perfringens* at d22 but not at d28 of the experiment.

On the other hand, although the up-regulation of *HSPs*, in particular *HSP70*, as well as *AvBD*, are considered to be a protective mechanism^47,48^ our results showed lower *HSP70* gene expression and no *AvBD* gene expression variation in NE birds. A possible explanation is that expression patterns of *AvBD* are pathogen-dependant, and AvBD8, 10, 11, and 13 are defensins that may play a key role in host intestinal defence against NE pathogens^49^. Nonetheless, none of these was incorporated in our gene expression panel.

### Oxidative stress markers and digestive hormones

Several studies reported an occurrence of OX in chickens challenged with *Eimeria*^40,50^ and *C. perfringens*^51^ evidenced by reduced antioxidant enzyme activity. However, our results showed that although *Eimeria* challenged chickens showed an up-regulation of *HIF1A* and *XDH,* as well as a tendency to increase *SOD1*, *C.perfringens* challenge decreased *SOD1* and *HMOX2* mRNA levels. These differences confirm that the damage caused was more pronounced in case of C*.perfringens* challenge, where a decrease of oxidative enzymes indicates a failure of these chickens to combat the excess free radicals produced during the infection as well as to promote an anti-inflammatory response as proven by the reduced mRNA levels of *GHRL*^52^*.* In fact, *GHRL* gene expression was up-regulated in duodenum and jejunum of heat-stressed^53^ and bursa of IBDV-infected broiler chickens^52^, which suggests that *GHRL* may function as an anti-inflammatory factor^52^.

In conclusion, the panel developed in the current study allows a global gene expression profiling which gives a greater overview of genes and pathways involved in broiler response to pathogen challenge. It also provides insights into differences of gene expression patterns and magnitude of responses under either a coccidial vaccine challenge or NE induced by the use of commercial reused litter and wheat-based diets without exogenous supplementation of enzymes. Considering these results, further studies will be performed using this panel to explore the underlying molecular mechanisms responsible of positive effects of feed additives containing organic acids and essential oils on broiler chickens gut health and performance.

## Material and methods

### Ethics approval and consent to participate

All animal experimentation procedures were approved by the animal Ethics Committee of the Universitat Autònoma de Barcelona and were performed in accordance with the European Union guidelines for the care and use of animals in research (Council OFTHE. 20.10.2010. Off J Eur Union. 2010; 33–79), as well as the ARRIVE guidelines^54^.

### Feeding program, bird management and husbandry

#### C. perfringens challenge

A total of 148 1-d-old Ross 308 male broiler chickens were included in the study. Birds were obtained from a commercial hatchery, where they were vaccinated *in ovo* according to the standard vaccination program, against Marek disease, Gumboro disease and infectious Bronchitis. Nonetheless, all chicks used in the trial did not receive the coccidiosis vaccination.

Upon arrival, chicks were weighed and randomly assigned according to initial BW into two rooms, healthy conditions with 7 replicates (4 chicks per cage) *vs* challenging conditions with 10 replicates (12 chicks per pen) in order to get a similar initial average BW for each replicate. Both rooms share the same environmental conditions. Mean room temperature was maintained to 35 °C during the first four d post placement and then decreased progressively to 25 °C on d 14. The range of relative humidity was maintained between 50 and 70%. Light intensity and day-length were adjusted according to the producer recommendations. For the first two ds, birds were given 24 h of light which were reduced to 23 h of light and 1 h of dark from d3 to d10 and 18 h of light and 6 h of dark from d11 till the end of the experimental period. The birds were weighed by cage or pen, and feed intake was recorded at the end of each phase (10, 28 and 42 ds).

Chickens were given a 3-phase feeding program consisting of starter (0 to 10 d), grower (11 to 28 d) and finisher (29 to 42 d) phases. Supplementary Table 7 lists the composition of the antibiotic-free and coccidiostat-free basal diet used during each phase. All diets were formulated to meet the requirements for maintenance and growth for broilers (Fundación Española para el Desarrollo de la Nutrición Animal, 2008). Feed in mash form and water were available ad libitum. All diets used were sampled and stored for their subsequent analysis.

#### Eimeria challenge

A total of 140 one-d-old Ross 308 broiler male chickens were included in the experiment. The chicks shared the same floor pen during the first week. They also shared the same mash basal diet whose composition was similar to that used in the first phase of the experiment 1. On d 7, 120 birds were individually weighed and distributed into 30 battery brooders cage (4 chicks per cage) with the aim to get a similar initial average body weight for each cage. Dietary treatments were as follows: (1) the same basal diet used during the first week as negative control (NC) and (2) NC supplemented with (0.0033%) robenidine hydrochloride.

### Challenge procedure

#### C. perfringens challenge

To promote challenging condition to the animals, the floor area of the pen (1.5 × 0.75 m) was covered with a litter consisting of 10% of clean wood shavings and 90% of reused litter material. The reused litter material was obtained from a commercial poultry flock where a clinical NE was claimed as previously characterized for its content of mesophilic aerobic bacteria (> 10^5^/g), Enterobacteriaceae (520 × 10^2^/g), filamentous fungi and yeasts (220 × 10^2^/g), sulphite-reducing anaerobes and *Clostridium perfringens* (> 10^5^/g*)*. A challenging method consisting to expose broilers to litter contaminated by *Clostridium perfringens* has been previously described^8^. Moreover, percentages of 15, 20 and 25% of wheat were incorporated in the starter, grower and finisher diets, respectively, without exogenous enzyme supplementations with the aim to increase viscosity of intestinal digesta.

#### Eimeria challenge

On d 9 of the study, all birds were orally gavaged with a commercial coccidiosis vaccine (EVANT, HIPRA, Spain) containing a mixture of viable sporulated oocysts (3.5 × 10^4^ *Eimeria acervulina*, 2.1 × 10^4^ *Eimeria maxima*, 3.1 × 10^4^ *Eimeria praecox*, 3.1 × 10^4^ *Eimeria mitis* and 2.9 × 10^4^ *Eimeria tenella*). This amount was chosen to represent a strong coccidia challenge that would induce intestinal damage, but not result in bird mortality. Housing system was also taken into account as chickens were reared in brooder cages where recycling of live oocysts by reinfection from litter is not possible as in floor pens^30^.

### Growth performance and sample collection

#### C.perfringens challenge

The chickens were weighed, and feed disappearance was determined at 0, 10, 28 and 42 ds of age. Mortality rate and BW of dead birds were also daily recorded. From these values, average daily feed intake (ADFI), ADG, and FCR corrected by mortality were calculated. On d 42, one bird per replicate was euthanized and samples were taken for ileum histomorphology analysis and ileum gene expression.

#### Eimeria challenge

To study the effect of both *Eimeria* challenge and coccidiostat supplementation sampling was performed on d 7 (pre-challenge) and 15 (post-challenge). All extra chickens (20) were euthanized on d 7 and one bird per replicate was euthanized on d 15 to collect ileum tissue for gene expression analysis and jejunum mucosa for proteomics analysis.

On d 15, faeces samples from all replicates were also collected for *Eimeria* oocyst count.

### Performed analyses

#### Eimeria oocyst count

Faecal samples were sent to the Laboratory of Parasitology Service. The oocyst counting was conducted using the McMaster egg-counting technique according to Roepstorff and Nansen, 1998. Briefly, four grams of faecal sample were transferred into a container, 56 ml of tap water were added, and the material was mixed thoroughly with a stirring device to ensure uniform suspension. Then faecal suspension was poured through a tea strainer and a 10-ml tube was filled to capacity with the filtered suspension. The tube was centrifuged for 2 min at 1800 RPM, the supernatant was removed and 4 ml of Zinc sulphate solution (SO_4_Zn 33%, 1.18) was added. The sediment was then carefully resuspended and the McMaster counting chamber was filled. The number of oocyst per gram of faeces was calculated by multiplying the total number of oocysts by a coefficient of 20.

#### Intestinal morphological analyses

Ileum samples of about 5 cm were collected at the midpoint between Meckel’s diverticulum and the ileo-cecal junction. Tissue sections (5 μm) were fixed in 4% paraformaldehyde and then embedded in paraffin. Afterwards, the sections were prepared, stained with haematoxylin eosin and analysed using a light microscope. The morphometric variables measured included villus height (VH), crypt depth (CD), the ratio of villus height to relative crypt depth (VH:CD) and intraepithelial lymphocytes (per 100 µm villus height). For the determination of goblet cell number (per 100 µm villus height), tissue slides were prepared and stained with periodic acid-Schiff. Ten villi were measured for each sample and only complete and vertically oriented villi were evaluated. The mean from 10 villi per sample was used as the average value for further analysis. All morphometric analysis was done by the same person, who was blinded to the treatments.

#### Tissue collection, RNA purification and cDNA synthesis

At the midpoint of ileum samples of about 1 cm were collected, snap frozen in 1 mL of RNA later (Deltalab, Spain) and stored at  − 80 °C for subsequent RNA isolation and gene expression analysis.

A sample of 50 mg of ileum tissue was submerged in 1 mL of TRIzol Reagent (Thermo Fisher Scientific) and homogenized with a Polytron device (IKA, Staufen, Germany). Total RNA was obtained with the Ambion RiboPure kit (Thermo Fisher Scientific), by following the manufacturer’s protocol. RNA concentration was measured with a NanoDrop ND-1000 spectrophotometer (NanoDrop products) and RNA purity was checked with Agilent Bioanalyzer-2100 equipment (Agilent Technologies), according to the producer’s protocol. All samples showed an RNA integrity number higher than 8.

Between 0.8 and 1 µg of total RNA was reverse-transcribed into cDNA with random primers using the High-Capacity cDNA Reverse Transcription kit (Applied Biosystems) in a final volume of 20 µl. The following thermal profile was applied: 25 °C 10 min; 37 °C 120 min; 85 °C 5 min; 4 °C hold. A negative control was performed with no reverse transcription (−RT control) to test the possible residual genomic DNA amplification. cDNA samples were stored at  − 20 °C until use.

#### Primer design and testing

Genes included in this study were previously selected based on published information. Primers were designed for 48 genes using the PrimerExpress 2.0 software (Applied Biosystems) (Supplementary Table 8). Primers were designed spanning exon-exon boundaries or alternatively located at different exons. In addition, genomic DNA amplification and primer dimer formation were controlled.

All primers were tested for quantitative real-time polymerase chain reaction (RT-qPCR) performance and specificity in an ABI PRISM 7900 Sequence Detection System (Applied Biosystems) using two-fold serial dilutions (1/10, 1/100) of a pool of cDNA from all samples. −RT controls were also included.

#### Selection of genes for the expression panel

A list of 48 genes related to intestinal response to different environmental or dietary challenges was selected according to the bibliography^13,14,18^, among others. The list included: (1) genes participating in the BF (*CLDN1*, *FABP2*, *FABP6*, *JAM2*, *JAM3*, *MUC13, MUC2*, *OCLN*, *TJP1*, and *TJP2*); (2) a gene coding for an hormone involved in energy homeostasis (*GHRL*); (3) genes that play an important role for the IR like pattern recognition receptors, host defense peptides, cytokines and stress proteins (*AHSA1*, *AvBD6*, *AvBD9*, *HSPA4*, *IFNG*, *IL10*, *IL18*, *IL1B*, *IL22*, *IL4*, *IL8*, *NOS2*, *PTGES*, *TLR2*, *TLR4*, and *TNF*); (4) genes involved in MB processes (*COX16*, *EIF4EBP1*, *mTOR*, and *RPS6KB1*); (5) genes coding for NT such as solute carriers among others (*SLC15A1*, *SLC1A1*, *SLC1A4*, *SLC2A2*, *SLC34A2*, *SLC3A1*, *SLC5A1*, *SLC7A7*, and *VDR*); (6) in addition genes implicated in OX were selected (*GPX7*, *HIF1A*, *HMOX2*, *SOD1*, and *XDH*); and finally three avian reference genes were selected according to the bibliography (*LBR, NDUFA*, and *YWHAZ*) (Table 3).

**Table 3:** Summary of the bibliography used for the selection of the genes included in the panel and a brief description of their main function.

Function	Gene	Description	References
Barrier function	CLDN1	Transmembrane protein of TJ	^55^
	FABP2	Related with epithelial cell content and occurance	^23,55^
	FABP6	Necessary for the transport of bile acids in the gut and it was associated with bacterial presence and inflammation	^55^
	JAM2	Transmembrane protein of TJ	
	JAM3	Transmembrane protein of TJ	
	MUC13	Transmembrane mucine that plays a role in cell signaling pathways	^23^
	MUC2	Secretory mucine important in the establishment of the mucus layer	^23,55^
	OCLN	Protein of TJ involved in both inter-membrane and paracellular diffusion of small molecules	
	TJP1	Scaffold proteins that form part of the cytoplasmatic plaque of TJ	^55^
	TJP2	Scaffold proteins that form part of the cytoplasmatic plaque of TJ	
Digestive hormone	GHRL	Induces motor activity in the intestinal tract	^52,53^
Immune response	AHSA1	Co-chaperone activator of HSP90	^12^
	AvBD6	Avian defensin involved in antimicrobial functions and protecting the gut epithelium	^46,56,57^
	AvBD9	Avian defensin with antimicrobial properties and other cellular functions	
	HSPA4	Member of HSP proteins and play a prominent role in repair and protection of the intestinal environment	^48,58,59^
	IFNG	Host defense for combating against the intracellular pathogens including Salmonella	^57,60,61^
	IL10	Anti-inflammatory cytokine produced by activated macrophages and T cell	^57,60,61^
	IL18	Pro-inflammatory cytokine, primarily produced by macrophages, targeting T helper type-1 (Th1) cells	^48,57,61,62^
	IL1β	Mediator of the inflammatory response and involved in cellular processes	^37,60^
	IL22	Commonly used as marker of inflammation involved in T-lynphoocytes activation	^37,60^
	IL4	Cytokine that induces differentiation of naive helper T cells (Th0 cells) to Th2 cells	^57,59,61^
	IL8	Secreted in response to pathogenic bacteria infection or specific inflammatory citokines	^57,58,61,63^
	NOS2	Induce the development of Th1 type of IR in infections	^37,60^
	PTGES	Intestinal inflammatory factor	^64^
	TLR2	Transmembrane receptor for the recongition of gram positive bacteria	^47,49,60,61^
	TLR4	Transmembrane receptor for the recongition of gram negative bacteria	
	TNFa	Regulation of the host immunity against multiple pathogens	^57,61^
Metabolism	COX16	Enzyme involved in the generation of energy by the mitochondria	^65^
	*EIF4EBP1*	mTOR pathway proteins in jejunum—protein synthesis and cell proliferation	^66,67^
	mTOR	mTOR pathway proteins in jejunum—protein synthesis and cell proliferation	
	RPS6KB1	mTOR pathway proteins in jejunum—protein synthesis and cell proliferation	
Nutrient transport	SLC15A1	Peptide transporter	^13^
	SLC1A1	Excitatory amino acid transporter	^68–71^
	SLC1A4	Neutral amino acid transporter by ASC system	^71^
	SLC2A2	Glucose transporter	^70–72^
	SLC34A2	n Intestinal phosphate absorption and phosphate homeostasis	^66^
	SLC3A1	Protetin related to neutral amino acid transporter	^13^
	SLC5A1	Sodium glucose transporter 1	
	SLC7A7	L amino acid transporter 2	
	VDR	Transcription factor that mediates the vitamin D3, involved in signaling intestinal calcium and phosphate absorption	^65,67,73^
Oxidation	GPX7	Intracellular antioxidant, and plays a great role in the detoxification of various peroxides	^73^
	HIF1A	Transcription factor that regulates genes involved in inflammation and cell death	^23^
	HMOX2	Oxidative stress marker	^74^
	SOD1	Antioxidant enzyme	^73^
	XDH	Enzyme associated to the synthesis of reactive oxygen species and is member of cellular defense system	^23^
Reference gene	LBR	Reference gene	^75^
	NDUFA	Reference gene	
	YWHAZ	Reference gene	^76^

#### Gene expression study in chicken intestine

RT-qPCR efficiency was checked using relative standard curves which were constructed for each gene assay with three-fold serial dilutions of the pool of cDNA and it was analysed per triplicate. The dilution series consisted of 1/5, 1/15, 1/45, 1/135, and 1/405 dilutions of pooled cDNA. High throughput RT-qPCR was performed in a 48.48 microfluidic dynamic array IFC chip in a BioMark HD System (Fluidigm Corporation)^77^. After that, mRNA quantification of 48 genes (45 target genes and 3 reference genes) of 15 animals was performed per duplicate using the 1/20 final dilution and a negative control was included to check the non-specific amplification of primers.

#### Protein expression

Approximately 30 mg of mucosa was homogenized (Polytron, Kinematica AG, Luzern, Swiss) in phosphate saline buffer at 4 °C during 30 s. Then, the samples were submitted to 3 freeze–thaw cycles (3 min each cycle) and centrifuged (3500 g, 10 min, 4 °C). The supernatant was used to quantify OCLN and CLDN1 by ELISA following the instructions of the manufacturer (Chicken occludin ELISA Kit and chicken claudin 1, MyBioSource Inc., San Diego, CA, USA).

#### Statistical analysis

The performance data were analysed considering the pens of birds as the experimental unit. All data concerning ADG, ADFI, FCR and intestinal histology from *C.perfringens* challenge experiment as well as BW from *Eimeria c*hallenge were analysed as one-way ANOVA using the GLM procedure for the statistical package SAS. Data of oocyst counting and protein expression were analyzed with T-test. When significant treatment effects were disclosed, statistically significant differences among means were determined by a Tukey multiple comparison test of means. The level of statistical significance was set at *P* ≤ 0.05 and tendency at *P* ≤ 0.10. Protein expression analysis was performed.

##### Processing, normalization, and statistical analysis of the RT-qPCR data

Data was collected and checked with Fluidigm Real-Time PCR analysis software 3.0.2 (Fluidigm) and analysed using DAG Expression software 1.0.4.11^78^. The relative standard method curve was applied; target gene expression levels were normalized using reference genes and resulted in normalized quantity (NQ) values of each sample and assay. Statistical computations were performed using R 3.4.3 (R Development Core Team 2013). Firstly, all data were subjected to a logarithmic transformation to get closer to the Gaussian distribution. The gene expression data were subjected to one-way ANOVA using treatment as factor. The differences between treatments averages associated to *P*-values ≤ 0.05 were regarded as statistically significant.

The Benjamini and Hochberg FDR^7^ multiple testing correction was added to the initial *P*-value information. The mean profiles of the treatments throughout the genes have been represented using a lines graph.

Two visualization tools based on unsupervised statistical learning methods were performed: PCA and heatmap representation. PCA for dimension reduction and visualization was applied to samples as cases and genes expressions as variables. Heatmaps are frequently used to represent expression levels and to show a double clustering for both samples and genes. In our setting, different clustering methods were used for samples (columns) and genes (rows). Indeed, a correlation-based distance $d = \left( {1 - r} \right)/2$, where *r* is the Pearson’s coefficient, and the complete linkage method were chosen to classify genes proximities, while the usual Euclidean distance and the Ward’s linkage were implemented to conglomerate the samples^79^.

## Supplementary Information

Supplementary Information

## Data Availability

All data generated or analysed during this study are included in this published article.
