# Supplementary material for: Transversal gene expression panel to evaluate intestinal health in broiler chickens in different challenging conditions
Source: Sci Rep. 2021 Mar 18;11:6315. doi: 10.1038/s41598-021-85872-5 (PMC7973573; doi:10.1038/s41598-021-85872-5)

Transversal gene expression panel to evaluate intestinal health in broiler chickens in different challenging conditions

Criado-Mesas, L.[1,¥,*], Abdelli, N.[2,¥], Noce, A.[3,4], Farré, M.[5], Pérez, J.F.[2], Solà-Oriol, D.[2], Martin-Venegas, R.[6,7], Forouzandeh, A.[2], González-Solé, F.[2], and Folch, J.M.[1,3]

[1]Plant and Animal Genomics, Centre for Research in Agricultural Genomics (CRAG), CSIC-IRTA-UAB-UB Consortium, Bellaterra, Spain.

[2]Animal Nutrition and Welfare Service, Animal and Food Science Department, Facultat de Veterinària, Universitat Autònoma de Barcelona, 08193 Bellaterra, Spain.

[3]Animal and Food Science Department, Facultat de Veterinària, Universitat Autònoma de Barcelona, 08193 Bellaterra, Spain.

[4]Leibniz-Institute for Farm Animal Biology (FBN), Wilhelm-Stahl Allee 2, 18196 Dummerstorf Germany.

[5]Department of Mathematics, area of Statistics and Operations Research, Universitat Autònoma de Barcelona, 08193 Bellaterra, Spain.

[6]Department of Biochemistry and Physiology, Facultat de Farmàcia i Ciències de l'Alimentació, Universitat de Barcelona, 08028 Barcelona, Spain.

[7]Research Institute of Nutrition and Food Safety (INSA-UB), Universitat de Barcelona, 08291 Santa Coloma de Gramanet, Spain.

[¥] Equally contributing authors

***Corresponding author:** Criado-Mesas, L.

E-mail: lourdes.criado@cragenomica.es

Supplementary Table 1. Effect of *C.perfringens* challenge on growth performance of broiler chickens.

Items	Conditions		SEM	*P*-value
	Cages	Challenge		
BW, g				
0 d	43.8	43.6	0.11	0.151
10 d	246.1^{a}	228.4^{b}	6.17	0.044
28 d	1484.9^{a}	1020.0^{b}	31.14	<0.001
42 d	2822.4^{a}	1912.1^{b}	56.27	<0.001
ADG, g/d				
0-10 d	20.2	18.6	0.61	0.056
10-28 d	68.8^{a}	43.1^{b}	1.79	<0.001
28-42 d	95.5^{a}	67.2^{b}	2.40	<0.001
0-42 d	66.1^{a}	44.7^{b}	1.30	<0.001
ADFI, g/d				
0-10 d	31.6^{a}	26.3^{b}	0.75	<0.001
10-28 d	95.2	82.9	5.37	0.099
28-42 d	163.6	152.8	6.88	0.247
0-42 d	101.6	92.6	3.36	0.057
FCR				
0-10 d	1.31^{b}	1.71^{a}	0.062	<0.001
10-28 d	1.38^{b}	1.93^{a}	0.114	0.002
28-42 d	1.71^{b}	2.30^{a}	0.121	0.002
0-42 d	1.54^{b}	2.07^{a}	0.052	<0.001

$^{(a,b)}$Values with different superscript letters indicate significant differences between groups ($P \leq 0.05$).

Supplementary Table 2. Effect of *C.perfringens* challenge on ileal morphological parameters of broiler chickens on day 42

Items	Conditions		SEM	*P*-value
	Cages	Challenge		
Villus height (µm)	933.1[a]	828.6[b]	29.43	0.016
Crypt depth (µm)	88.9[b]	219.6[a]	6.46	<0.001
Villus height/crypt depth (µm)	10.5[a]	3.77[b]	0.29	<0.001
Goblet cells/100 µm VH	11.6	15.8	1.29	0.139
Intraepithelial Lymphocytes/100 µm VH	2.3[b]	7.9[a]	0.45	<0.001

[(a,b)]Values with different superscript letters indicate significant differences between groups ($P \leq 0.05$).

Supplementary Table 3. Relative gene expression differences between broiler chickens raised either in cages or under *Clostridium perfringens* challenge conditions

Function	Gene	Experimental treatments		Contrast Statistic	*P*-value	FDR
		Cages	Challenge			
Barrier function	*CLDN1*	1.43	0.64	12.136	0.004	0.009
	FABP2	1.68	0.33	51.544	<0.001	<0.001
	FABP6	1.39	0.83	4.889	0.045	0.077
	JAM2	1.45	1.1	1.276	0.279	0.361
	JAM3	0.99	0.95	0.011	0.986	0.986
	MUC13	2.32	0.37	12.857	0.003	0.009
	MUC2	1.36	0.83	8.933	0.01	0.022
	OCLN	1.67	0.65	34.891	<0.001	<0.001
	TJP1	1.57	0.81	50.084	<0.001	<0.001
	TJP2	1.37	0.84	15.35	0.002	0.005
Digestive hormone	*GHRL*	1.23	0.69	5.649	0.033	0.061
Immune response	*AHSA1*	1.41	1.12	1.348	0.266	0.355
	AvBD6	0.94	0.58	0.819	0.382	0.445
	HSPA4	1.34	1.06	4.965	0.044	0.077
	IFNG	0.24	1.79	82.337	<0.001	<0.001
	IL10	0.05	1.61	226.069	<0.001	<0.001
	IL18	0.56	1.43	45.503	<0.001	<0.001
	IL1B	0.55	1.19	9.582	0.009	0.02
	IL22	0.34	2.3	18.752	0.001	0.002
	IL4	2.45	2.46	0.888	0.363	0.444
	IL8	0.4	1.68	45.243	<0.001	<0.001
	NOS2	0.82	1.3	2.855	0.115	0.158
	PTGES	0.83	0.9	0.143	0.711	0.745
	TLR2			7.153	0.019	0.038
	TLR4	1.43	1.16	0.894	0.362	0.444
	TNFα	1.43	0.96	22.629	<0.001	0.001
Metabolism	*COX16*	1.28	1.06	5.933	0.03	0.057
	EIF4EBP1	1.07	0.76	3.985	0.067	0.101
	mTOR	1.27	0.87	19.79	0.001	0.002

	RPS6KB1	1.31	0.98	4.458	0.055	0.089
Nutrient transport	*SLC15A1*	0.87	0.68	0.812	0.384	0.445
	SLC1A1	1.57	0.6	49.909	<0.001	<0.001
	SLC1A4	1.72	0.69	33.85	<0.001	<0.001
	SLC2A2	1.19	0.81	4.227	0.06	0.095
	SLC34A2	1.3	0.96	9.012	0.01	0.022
	SLC3A1	1.01	1.3	3.8631	0.072	0.101
	SLC5A1	1.07	0.85	3.8497	0.072	0.101
	SLC7A7	0.87	1.11	0.5939	0.455	0.468
	VDR	1.49	0.6	26.0385	<0.001	0.001
Oxidation	*GPX7*	1.03	1.31	0.7593	0.399	0.45
	HIF1A	1.05	0.97	0.5694	0.464	0.498
	HMOX2	1.46	0.82	41.0743	<0.001	<0.001
	SOD1	1.55	0.69	38.5423	<0.001	<0.001
	XDH	1.03	1.01	0.0247	0.877	0.898

Supplementary Table 4. Effect of dietary treatments on BW of broiler chickens before (d7 and 9) and after Eimeria challenge (d15)

	NC	NC+coccidiostat	SEM	*P*-value
BW7	158	158	0.92	0.99
BW9	220	223	1.9	0.62
BW15	383^{b}	464^{a}	4.3	<0.001

Supplementary Table 5. Relative gene expression differences between broiler chickens receiving the negative control diet before and after the challenge

Function	Gene	Experimental treatments		Contrast Statistic	*P*-value	FDR
		Before challenge	After challenge			
Barrier Function	CLDN1	8.43	2.66	16.1921	<0.001	0.007
	FABP2	3.73	3.61	0.0034	0.954	0.954
	FABP6	4.2	2.66	13.5261	0.001	0.01
	JAM2	3.67	4.77	10.1648	0.004	0.019
	JAM3	3.74	4.37	4.1972	0.051	0.132
	MUC13	1.09	2.04	2.4192	0.132	0.257
	MUC2	4.24	3.9	0.2438	0.626	0.744
	OCLN	3.69	4.26	2.949	0.098	0.206
	TJP1	3.6	4.33	11.326	0.002	0.014
	TJP2201	3.58	3.77	0.8324	0.37	0.537
Digestive hormone	GHRL	4.73	6.22	3.2513	0.083	0.193
Immune response	AHSA1	3.85	3.75	0.1736	0.68	0.768
	AVBD6	5.07	2.45	1.907	0.18	0.317
	HSPA4	3.5	4.08	11.8148	0.002	0.013
	IFNG	1.34	5.1	52.3817	< 0.001	< 0.001
	IL1b	3.99	4.35	0.9611	0.336	0.51
	IL18	4.01	3.9	0.0065	0.936	0.954
	IL8	7.76	4.61	2.3922	0.135	0.257
	IL10	3.01	4.64	4.9098	0.036	0.106
	IL22	5.22	4.36	0.0545	0.817	0.877
	IL4	2.65	4.56	2.1634	0.156	0.286
	NOS2	3.53	4.62	6.2806	0.019	0.07
	PTGES	3.84	4.26	1.3449	0.257	0.419
	TLR2	3.41	3.93	3.5192	0.072	0.177
	TLR4	4.53	4.68	0.208	0.652	0.755
	TNFa	4	3.74	0.735	0.399	0.549
Metabolism	EIF4EBP1	3.8	3.93	0.5748	0.455	0.572
	COX16	4.27	4.24	0.0085	0.927	0.954
	mTOR	3.66	4.04	6.0078	0.022	0.073
	RPS6KB1	3.79	4.16	8.6725	0.007	0.03
	SLC1A4	4.24	3.96	1.2647	0.271	0.427

Nutrient transport	SLC5A1	3.81	3.72	0.1452	0.706	0.777
	SLC15A1	2.71	4.7	15.2767	<0.001	0.007
	SLC1A1	3.74	3.18	1.3593	0.255	0.419
	SLC2A2	5.39	2.05	6.8316	0.015	0.06
	SLC34A1	3.52	3.88	0.8044	0.378	0.537
	SLC34A2	4.41	3.52	0.5873	0.451	0.573
	SLC7A7	5.07	4.44	0.5219	0.477	0.583
	VDR	3.52	4.25	4.408	0.046	0.127
Oxidation	GPX7	3.91	3.65	0.6674	0.422	0.562
	HIF1a	3.33	3.99	12.1811	0.002	0.013
	HMOX2	3.93	4.33	3.0336	0.094	0.206
	SOD1	3.43	4.58	5.3382	0.029	0.092
	XDH	2.83	4.8	28.8664	< 0.001	< 0.001

Supplementary Table 6. Relative gene expression differences between broiler chickens fed the negative control diet and those supplemented with coccidiostat

Function	Gene	Experimental treatments		Contrast Statistic	P-value	FDR
		NC	NC+ coccidiostat			
Barrier function	CLDN1	2.66	2.99	0.8113	0.378	0.573
	FABP2	3.61	4.34	2.7035	0.115	0.316
	FABP6	2.66	3.04	1.5408	0.228	0.441
	JAM2	4.77	5.96	6.9324	0.016	0.168
	JAM3	4.37	5.02	4.5323	0.045	0.173
	MUC13	2.04	1.08	4.4434	0.047	0.173
	MUC2	3.9	4.51	3.9469	0.06	0.192
	OCLN	4.26	4.17	0.0476	0.83	0.942
	TJP1	4.33	4.92	4.5548	0.045	0.173
	TJP2201	3.77	4.25	5.7458	0.025	0.17
Digestive hormone	GHRL	6.22	6.58	0.0447	0.835	0.942
Immune response	AHSA1	3.75	3.31	5.6509	0.027	0.17
	AVBD6	2.45	4.13	1.2902	0.269	0.48
	HSPA4	4.08	4.16	0.113	0.74	0.942
	IFNG	5.1	2.68	21.7646	<0.001	0.006

	IL1b	4.35	3.89	0.9355	0.345	0.541
	IL18	3.9	4.49	2.0069	0.171	0.377
	IL8	4.61	5.24	1.0535	0.316	0.535
	IL10	4.64	3.48	2.5605	0.125	0.322
	IL22	4.36	6.29	1.2702	0.272	0.48
	IL4	4.56	1.67	4.9237	0.038	0.173
	NOS2	4.62	4.57	0.0006	0.98	0.992
	PTGES	4.26	4.15	0.0679	0.797	0.942
	TLR2	3.93	3.98	0.0044	0.948	0.992
	TLR4	4.68	5.31	3.7774	0.066	0.192
	TNF	3.74	3.3	4.8711	0.039	0.173
Metabolism	EIF4EBP1	3.93	3.96	0.0001	0.992	0.992
	COX16	4.24	4.17	0.0852	0.773	0.942
	mTOR	4.04	4.07	0.0335	0.857	0.942
	RPS6KB1	4.16	4.33	0.9945	0.33	0.538
Nutrient transport	SLC1A4	3.96	4.53	6.4506	0.019	0.168
	SLC5A1	3.72	3.78	0.0444	0.835	0.942
	SLC15A1	4.7	4.94	0.2814	0.601	0.827
	SLC1A1	3.18	4.33	9.7792	0.005	0.075
	SLC2A2	2.05	1.86	0.0161	0.9	0.966
	SLC34A1	3.88	4.66	2.2767	0.146	0.339
	SLC34A2	3.52	3.11	0.7228	0.405	0.594
	SLC7A7	4.44	6.21	9.7764	0.005	0.073
	VDR	4.25	4.57	0.5495	0.467	0.662
Oxidation	GPX7	3.65	4.32	3.7764	0.066	0.192
	HIF1a	3.99	4.27	1.7039	0.206	0.431
	HMOX2	4.33	4	2.407	0.136	0.332
	SOD1	4.58	4.24	1.5262	0.23	0.441
	XDH	4.8	4.92	0.2369	0.632	0.842

Supplementary Table 7. Basal diet composition.

	Starter		Growing		Finishing	
	Cages	Challenge	Cages	Challenge	Cages	Challenge
Ingredient composition, g/kg diet						
Maize	550	412	582	403	632	405
Wheat	***	150	***	200	***	250
Soybean meal 48	303	288	350	326	282	255
L-lysine HCL	1.2	1.5	0.2	0.7	1	1.6
DL-methionine	2.4	2.3	1.3	1.3	1.5	1.4
L-threonine	***	***	***	***	0.2	0.5
Soy oil	8	11	19	14	***	***
Palm oil	***	***	17	25	57	60
Enersoy-3600 (3002)	100	100	***	***	***	***
Limestone	10.3	10.9	6.3	7	5.8	7
Dicalcium phosphate	15.7	14.9	15.5	14.4	11.8	10.8
Salt	2	2	2	2	2.1	2
Premix*	4	4	4	4	4	4
Sodium bicarbonate	3.4	3.4	2.7	2.6	2.6	2.7
Calculated composition (%)						
Dry matter	87.8	88.1	87.9	88.1	88	88.4
M.E (kcal/kg)	2975	2975	3101	3101	3248	3249
Crude protein	22	22	21	21	18	18
Lysine	1.35	1.35	1.18	1.18	1.06	1.06

Methionine	0.59	0.58	0.47	0.46	0.45	0.43
Ca	0.95	0.95	0.78	0.78	0.65	0.67
Total P	0.65	0.64	0.63	0.62	0.54	0.53
Available P	0.45	0.45	0.44	0.44	0.37	0.37
Analyzed composition (%)						
Dry matter	88.5	90.2	88.2	90.4	88.3	90.6
GE, kcal/kg	4100	4081	4300	4332	4390	4395
Crude protein	20.9	20.5	21.4	21.3	17.9	18.5
Ether extract	0.43	0.48	0.54	0.6	0.76	0.79

Basal diet composition for broilers from 1 to 42 days (g/kg diet as fed-basis). (*) Provided per kg of feed: vitamin A (retinyl acetate) 10.000 UI; vitamin D3 (cholecalciferol) 4.800 UI; vitamin B1 (Thiamine) 3 mg; vitamin B2 (riboflavin) 9 mg; vitamin B3 (Nicotinamide) 51 mg; vitamin B6 (pyridoxine chlorhydrate) 4.5 mg; vitamin B9 (folic acid) 1.8; vitamin B12 (cyanocobalamin) 0.04 mg; vitamin E (acetate de tot-rac-3-tocopherol): 45 mg; vitamin K3 (Menadione) 3 mg; pantothenic acid (calcium D-pantothenate) 16.5 mg, biotin (D-(+)-biotin) 0.15 mg; Chloride of choline 350 mg; iron ($FeSO_4$) 54 mg; iodine ($Ca(IO_3)_2$) 1.2 mg; ; zinc (ZnO) 66 mg; manganese (MnO) 90 mg; copper ($CuSO_4$) 12 mg; selenium (Na_2SeO_3) 0.2 mg; 6-Phytase EC 3.1.3.26 : 1500 FYT; Butylated hydroxytoluene (BHT) 25 mg ; Colloidal silica 45 mg, Sepiolite 1007 mg

Supplementary Table 8. List of primers used for the analyses of gene expression of 48 genes by RT-qPCR.

Gene symbol	Gene Name	Gene Reference Sequence	Forward Primer (5’ -> 3’)	Reverse Primer (5’ -> 3’)
CLDN 1	Claudin-1	ENSGALG00000026862	CTTCATCATTGCAGGTCTGT CA	TTAACAGGTGTGAAAGGG TCATAGAA
FABP2	Fatty Acid Binding Protein 2	ENSGALG00000011986	GCTGACGGGACTGAACTTT CA	CGCTGTGAGTACTTTTCCA TTATCTTT
FABP6	Fatty Acid Binding Protein 6	ENSGALG00000001445	ACTATAGACAAGGAAGCA GACATGGA	TCCATTTTGACTGTTGCCT TGA
JAM2	Junctional Adhesion Molecule 2	ENSGALG00000015746	CATGAAGCGAATGCAAGTT GA	CCATTACCAGAGCCACAA TTACTACA
JAM3	Junctional Adhesion Molecule 3	ENSGALG00000001472	AGGAGCAGGAGATGGAAG TCTATG	TGTAGGCACAGCAGATAC CAAGAG

MUC13	Mucin-13	ENSGALG00000011806	GACTGATTTTGAAGTTCAGGAAGCA	CATTGGGATGTATTTGCGTACTG
MUC2	Mucin-2	ENSGALG00000031737	TGGAGGCAAAGTGTCTGCTCT	TAATAGCATGGGCATTTGGAGAT
OCLN	Occludin	ENSGALG00000037316	CTGCTCTGCCTCATCTGCTTCT	GCGCGGTCCCAGTAGATGT
TJP1	Tight Junction Protein 1	ENSGALG00000042552	CCATTTTTGGTCCAATAGCTGAT	CTCTCGGTTCACTTTTTGCAATT
TJP2	Tight Junction Protein 2	ENSGALG00000015109	TGAAATAGAGTCAAACAGATCGTTCTC	TATTGGATGGATCATCTTTTGCAT
GHRL	Ghrelin and Obestatin Prepropeptide	ENSGALG00000008411	GCTCTGGCTGGCTCTAGTTTTTTA	GGTTTTCTTGTATCCTTTTGTTGC
AHSA1	Activator Of HSP90 ATPase Activity 1	ENSGALG00000010463	TTGATGAAGTGGAGATCCTTGTCA	CCTTCTTGCTTCATCAGGGTCTT
AvBD6	Avian Beta-defensin 6	ENSGALG00000016668	CTTGCTGTGTGAGGAACAGGTG	TTTGGTAGTTGCAGGCAGGAT
AvBD9	Avian Beta-defensin 9	ENSGALG00000019845	TCCAGGCTGCTCCAGCTT	GGCTCTGCCTGCATGCTAA
HSPA4	Heat Shock Protein Family A (Hsp70) Member 4	ENSGALG00000040882	TGAGACTAATAAATGAATCAACTGCAGT	CCCCATATCCACAAAAACAACA
IFNG	Interferon Gamma	ENSGALG00000009903	GACAGAGAGAAATGAGAAAAGGATCA	CAGTGTTTTCAAGCATTTCCAAGT
IL10	Interleukin 10	ENSGALG00000000892	CTGAGGGTGAAGTTTGAGGAAAT	AGCCAAAGGTCCCCTTAAACTC
IL18	Interleukin 18	ENSGALG00000007874	CAGATTTAAACGTGGCAGCTTTT	CGAAGTACATTCCACTGCCAGAT
IL1B	Interleukin 1 Beta	ENSGALG00000029940	CGCTACACCCGCTCACAGT	GCAATGTTGAGCCTCACTTTCTG
IL22	Interleukin 22	ENSGALG00000009904	ACCTACACCTTGGCTGAAATGG	TGCTGACCGATGAGTCTGTTG
IL4	Interleukin 4	ENSGALG00000006827	TTATGCAAAGCCTCCACAATTG	GTGGGACATGGTGCCTTGAG
IL8	Interleukin 8	ENSGALG00000026098	CCACTGCTCCCTGGGTACAG	TCAGAATTGAGCTGAGCCTTG
NOS2	Nitric Oxide Synthase 2	ENSGALG00000038096	CTCCAGCAGAGCTTCTACCTCAA	GCCAGGTGCTCTTCTATTTTTAATTC
PTGES	Prostaglandin E Synthase	ENSGALG00000046708	TAAAGATGTATGTCGTTGCCATCAT	ATCCTCTGGGTTAGCAAATGCCT
TLR2	Toll Like Receptor 2	ENSGALG00000009239	AGGCGATCCCAAGAGGTTCT	TTTCCCAAAACATCTGCTGTTG
TLR4	Toll Like Receptor 4	ENSGALG00000007001	CAGTCCGTGCCTGGAGGT	TTGAGCTTAGCAATTTCAGACTGTTG
TNF	Tumor Necrosis Factor Alpha	ENSGALG00000003217	TGTTTCTGCCTCTGCCATCAG	AAGCCACTAGGAGCAGACATGATATAT
COX16	Cytochrome C Oxidase Assembly Homolog	ENSGALG00000020449	CCTGCTTTGAAGGAAAAATTGAAG	CCAAGTCAGATTGTTCCAATTTCTC
EIF4EBP1	Eukariotic initiation factor 4E binding protein	ENSGALG00000003205	ATTGAGAACAACCATGTCCAGAAC	ATGTCAAACTGCTCTTCTTCACCT
mTOR	Mechanistic Target of Rapamycin Kinase	ENSGALG00000003339	TGCTGACAAACGCTATGGAGGT	AGCCATGACACTGTCCTTATGCT
RPS6KB1	Ribosomal Protein S6 Kinase B1	ENSGALG00000005198	ACACCTGTTGATAGCCCAGATGA	GCCACATACGTAAAACCCAGAAA
SLC15A1	Solute Carrier Family 15 Member 1	ENSGALG00000016884	CCATGGAGTCAACAGGCTACAG	CTCACTCCCAAAATTTACCATTACAGAT
SLC1A1	Solute Carrier Family 1 Member 1	ENSGALG00000010187	CATTAAACCTGGAGTGCCTCAAA	GATCCAGCATGGCATCAACA
SLC1A4	Solute Carrier Family 1 Member 4	ENSGALG00000008811	CGAACGTATGCGACAGATTATAAGATAG	GTACCAACAGGGATCTTTTCCAA
SLC2A2	Solute Carrier Family 2 Member 2	ENSGALG00000009306	GAGGAGGCCAAAAAGAGTTTG	ACTCTCTTTTCACTCGCAGCTTCT
SLC34A2	Solute Carrier Family 34 Member 2	ENSGALG00000014372	AGAAGGAGAAAATCACAAAGGCA	TCAACGCTATTGTGGAAAATGC

SLC3A1	Solute Carrier Family 3 Member 1	ENSGALG00000009973	CTAGAAGCCACACATCTACGAGATGA	ATAGGCTGTGATGCTCTCAGGAT
SLC5A1	Solute Carrier Family 5 Member 1	ENSGALG00000006728	GGCAGTGGGAGTATGGGCTAT	GCTCCGTCCAGCCAGAAAG
SLC7A7	Solute Carrier Family 7 Member 7	ENSGALG00000034806	GCAAAACTTGTGGCCCTTGT	CTCTGAGGTTTTCTGTTTCTCCTTTA
VDR	Vitamin D Receptor	ENSGALG00000033090	CTGCAAAATCACCAAGGACAAC	CCTCGTCCGTCAGGATGAAC
GPX7	Glutathione Peroxidase 7	ENSGALG00000010633	GGTGCCTCCTTTCCTATGTTCA	GTTGGTTCTTCTCCAGTAGAATCAA
HIF1A	Hypoxia Inducible Factor 1 Subunit Alpha	ENSGALG00000011870	CACTTTTTCAGGCAGTTGGAATTG	TTTTGCACGCCTTTACACGTT
HMOX2	Heme Oxygenase 2	ENSGALG00000007638	TCCAGTCCACGATGGGAAA	GCATTGCCTGCTAGCTTGTCT
SOD1	Superoxide Dismutase 1	ENSGALG00000015844	CCGGCTTGTCTGATGGAGAT	CTGCGCTGGTACACCCATTT
XDH	Xanthine Dehydrogenase	ENSGALG00000008701	GAAGCCATTCCATTACTTCAGTTATG	AATGTCTGTGCGGATGTTCTTG
LBR	Lamin B Receptor	ENSGALG00000009305	CAGCAGATCCCAAATTGTCCTATC	CTGTGACAAGAAGCCCTTTTCC
NDUFA	NADH: Ubiquinone Oxidoreductase Subunit A1	ENSGALG00000008613	TGTGCAGAAACTACAGGACAAACTG	AGGGAAAGCTCATTTTCAGCCT
YWHAZ	Tyrosine 3-Monooxygenase/Tryptophan 5-Monooxygenase Activation Protein Zeta	ENSGALG00000031387	GCAAGCAGAAAGCAAAGTTTTCT	TGTGATTGCTCCACAATCCCT

Supplementary Figure 1. Oocyst counting in the excreta of chickens challenged with *Eimeria*

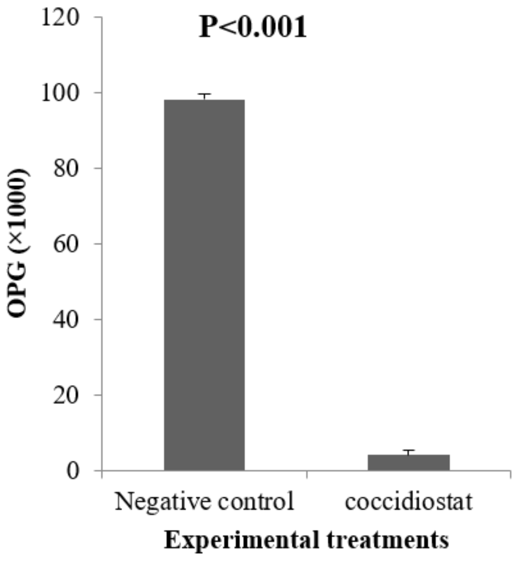

Supplement: Supplementary file 1 — Supplementary Information [file 41598_2021_85872_MOESM1_ESM.pdf]
